# Two new species of *Vertomannus* Distant, 1903 (Heteroptera, Rhyparochromidae, Ozophorini), with proposal of a new subgenus

**DOI:** 10.3897/zookeys.319.4323

**Published:** 2013-07-30

**Authors:** Katinka Varga, Előd Kondorosy

**Affiliations:** 1 Pannon University, Georgikon Faculty, Department of Animal Science, Deák F. Str. 16, H-8360 Keszthely, Hungary

**Keywords:** Heteroptera, Rhyparochromidae, Ozophorini, *Vertomannus*, new species, distribution, Oriental Region

## Abstract

Two new *Vertomannus* species, *Vertomannus flavus* **sp. n.** (south Vietnam) and *Vertomannus borneensis* **sp. n.** (Borneo) are described. *Vertomannus (Elongatomannus)*, **subgen. n.** (type species: *Vertomannus flavus*) is proposed.

## Introduction

Rhyparochromidae is the largest family of Lygaeoidea containing more than half of the described species of the superfamily. Species of the rhyparochromid tribe Ozophorini are distributed all over the world (Slater 1964; Slater and O’Donnell 1995). The diversity of Ozophorini is highest in tropical America and southeast Asia, however, the Oriental Region is the least investigated area.

The ozophorine genus *Vertomannus* Distant, 1903 was described as monotypic, containing *Vertomannus capitatus* Distant, 1903 from Assam and Burma (Distant 1903). Breddin (1905) described *Vertomannus tener* Breddin, 1905 from Java, but Bergroth (1916) subsequently transferred it to *Omacrus* Bergroth, 1916. Later Zheng (in Zheng and Zou 1981) described five new species from China and provided a key for the Chinese species. Thus, there are six described species of *Vertomannus* at present.

This paper contains descriptions of two new species of *Vertomannus* from Vietnam and Borneo. One of the new species is morphologically strikingly different from all other species of the genus: therefore, a new subgenus is proposed for its accommodation.

## Material and methods

Abbreviations for depositories: NHMW: Natural History Museum, Vienna, Austria; ZMAN: Zoological Museum, Amsterdam, The Netherlands; MMBC: Moravian Museum, Brno, Czech Republic.

Examination of the specimens was carried out using an ALPHA STO-4-65 Zoom and a Zeiss Disovery V8 stereomicroscopes. Measurements were taken using a micrometer eyepiece. Pictures were made using a Panasonic DMC G2 digital camera (12 Mpixel).

## Taxonomy

### Genus Vertomannus

Distant, 1903

http://species-id.net/wiki/Vertomannus

#### Redescription.

Body elongate. Head not punctured behind ocelli, posterior part slender, forming a long neck. Postocular part of head with neck longer than anteocular part. Eyes prominent. Ocelli situated close to eyes. Neck exserted. Antenna and legs slender, long. Antennal segment I and femora thickened apically. Fore femur often thick, always with spines. Pronotum with transversal furrow usually well developed, anterior and posterior lobes well separated. Anterior lobe of pronotum narrower than posterior one. Lateral margins of pronotum not flattened, humeri without spines. Middle of scutellum slightly elevated. Clavus with three or four rows of punctures. Scent gland ostiole with prominent and short peritreme curving posteriorly. Evaporatorium indistinct. Basal part of abdomen dorsoventrally constricted, not or weakly punctured. Intersegmental sutures between abdominal sterna 4 and 5 usually not curving cephalad, reaching lateral margin of abdomen.

**Discussion.** The genus *Vertomannus* is similar to *Cervicoris* Slater, 1982, but the latter genus is different in having the anterior lobe of the pronotum globose, and the presence of a pair of long humeral spines. Additionally, *Cervicoris* has the intersegmental sutures between abdominal sterna 4 and 5 curving cephalad, not reaching the lateral margin of abdomen, which is a typical rhyparochromid character.

### Descriptions of new taxa

#### Vertomannus (Elongatomannus) subgen. n.

urn:lsid:zoobank.org:act:AD5A59AD-B19E-4F92-ABA8-AD2F55C6AE4C

http://species-id.net/wiki/Vertomannus_(Elongatomannus)

##### Description.

Head elongate, anteocular part distinctly longer than postocular part without neck, nearly horizontal, weakly declivous, antennifers not closer to eyes than longitudinal diameter of eye. Head, especially neck, with sparse long and dense short pilosity, neck transversely wrinkled. Humeral angles of pronotum prominent, posterior lobe medially elevated. Apex of labium surpassing mid coxa. Scutellum triangular, with faint Y-shaped elevation. Clavus with four full rows of punctures.

##### Discussion.

The new subgenus can readily be separated from the nominotypical subgenus based on the above mentioned characters: all known members of *Vertomannus (Vertomannus)* have a globose head with only sparse long pilosity (without short hairs) and with a relatively short anteocular part, a smooth neck; humeral angles of pronotum not prominent, labium not surpassing fore coxa, scutellum without a distinct elevation and clavus with only three rows of punctures.

**Type species** (by present designation): *Vertomannus (Elongatomannus) flavus* sp. n.

##### Etymology.

Formed by the combination of the Latin prefix *elongato*- ‘elongate’ and –*mannus*, the former referring to the strikingly elongate head and neck characteristic for the subgenus, the latter referring to the genus *Vertomannus*. Gender masculine.

#### Vertomannus (Elongatomannus) flavus sp. n.

urn:lsid:zoobank.org:act:8687A7A6-CB96-4C94-BADF-C5ADB6981D24

http://species-id.net/wiki/Vertomannus_flavus

##### Type material.

Holotype: female, pinned. Original label: “S Vietnam, 14°10'N, 108°30'E 40km NW of An Khe, Buon Luoi, 650–750m 28.3.–12.4. 1995, Pacholátko & Dembicky leg.” [printed] (NHMW).

Paratype: 1 female, pinned. Original labels: “Laos, 24–29. IV. 2001, 18°07'N, 104°29'E, Khammouan pr. Ban Khoun Ngeun ca 200m, V. Kubáň lgt.”, “Collectio Petr Baňař, Moravian Museum Brno” (MMBC).

**Measurements** (in mm). Body length: 9.75; head length with neck: 2.75, width: 1.0, interocular space: 0.55, anteocular length: 0.7, postocular length: 0.55, neck length 1.15; pronotum: length: 1.5, humeral width: 1.55; scutellum: length: 1.0, width: 0.9; length of claval commissure: 2.0; lengths of antennal segments: I 0.95, II 2.9, III 2.35, IV 2.45; lengths of labial segments: 5.05 = I 1.5, II 1.6, III 1.4, IV 0.55.

##### Description.

**Figure 1.**
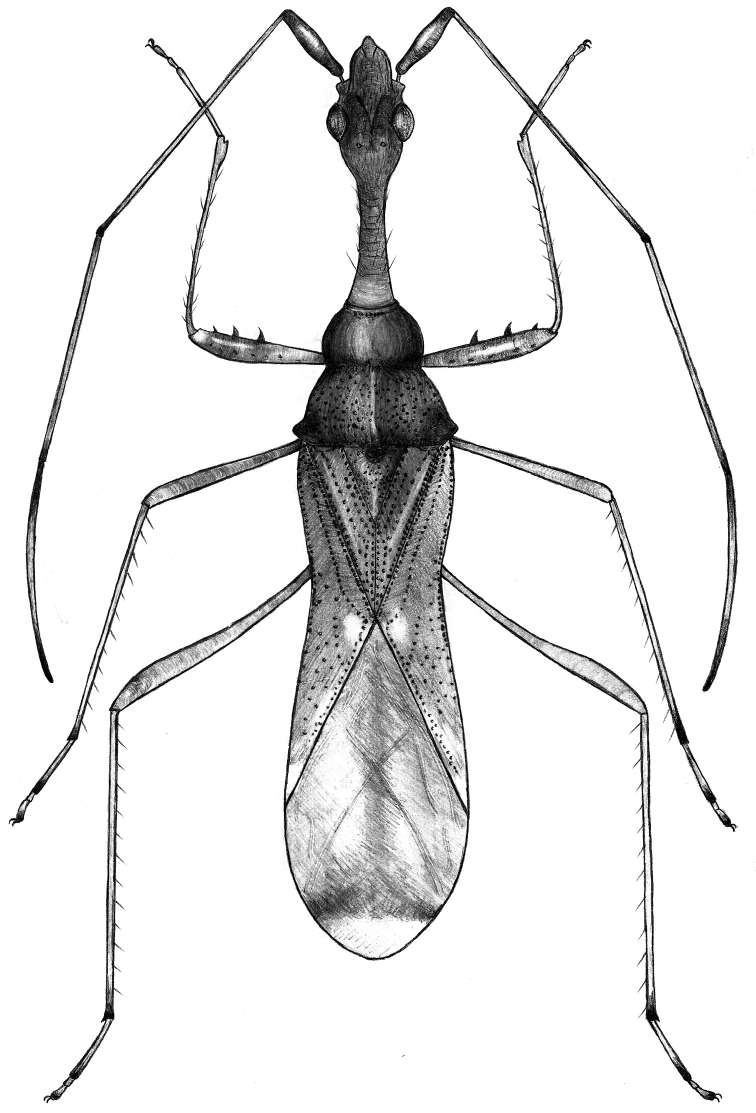
*Vertomannus (Elongatomannus) flavus* sp. n., dorsal view

**Figure 2.**
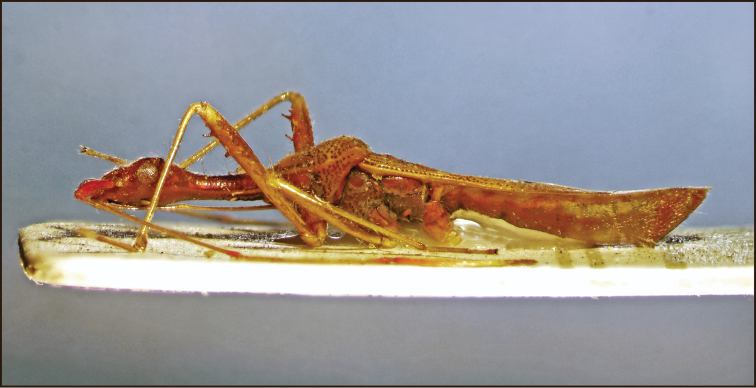
*Vertomannus (Elongatomannus) flavus* sp. n., lateral view.

Body elongate, dorsum moderately shiny, thoracic sternum dull (Fig. 1). Head, neck and ventral side of body with short semidecumbent hairs, long erect hairs present only on head (Fig. 2).

Head: head-neck transition very gradual. Antennal segment I thickened apically, remaining segments long and filiform.

Thorax: pronotum trapeziform, lateral margin concave, anterior lobe narrower than posterior, anterior lobe without punctures except at margins, posterior lobe distinctly punctured, punctures with very short inconspicuous setae, posterior lobe a little more declivous than anterior lobe in lateral view. Scutellum triangular, width and length subequal, with dense punctures except faint Y-shaped elevation, with very fine, hardly visible hairs.

Legs slender and long, fore femur distally with two large spines and one small spine, with hairs as long as diameter of femur. Corium less punctured, not pubescent. Abdomen: densely pubescent with silvery hairs.

**Colouration.** Body stramineous, punctures brown. Head and antennal segment I reddish brown, antennal segments II-III stramineous with reddish apex, segment IV brown with basal third reddish. Lateral margin of anterior lobe of pronotum darker than disk, punctures on posterior part brown. Scutellum slightly darker than ground colour of body. Membrane honey-coloured, apical part translucent, subapically with a transverse stripe connected with a longitudinal stripe.

##### Discussion.

The long and stramineous body and the features in the diagnosis of the subgenus easily separate this species from all known *Vertomannus* species.

##### Etymology.

The species is named after the unique colouration. The epithet “flavus” is a Latin adjective in nominative case, meaning “yellow”.

#### Vertomannus (Vertomannus) borneensis sp. n.

urn:lsid:zoobank.org:act:ADA68F97-E0A8-4A6D-9D06-9BF7813922E8

http://species-id.net/wiki/Vertomannus_borneensis

##### Type material.

Holotype: female, pinned. Original label: “C. Borneo, Long Nawang, leg. Mjöberg 1925” [printed] (ZMAN).

**Measurements** (in mm). Body length: 5.85; head length with neck: 1.63, width: 0.95, interocular space: 0.6; anteocular length: 0.3, postocular length: 0.5, neck length 0.53; pronotum: length: 0.95, width: 1.55; scutellum: length: 0.95, width: 0.6; length of claval commissure: 1.4; Length of antennal segments: I 0.75, II 1.8, III 1.5, IV missing; lengths of labial segments: 1.65 = I 0.5, II 0.35, III 0.5, IV 0.3

##### Description.

**Figure 3.**
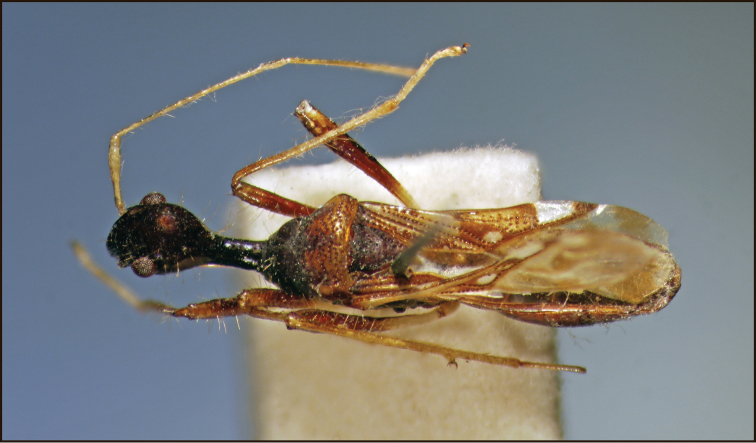
*Vertomannus* (s. str.) *borneensis* sp. n., dorsal view.

**Figure 4.**
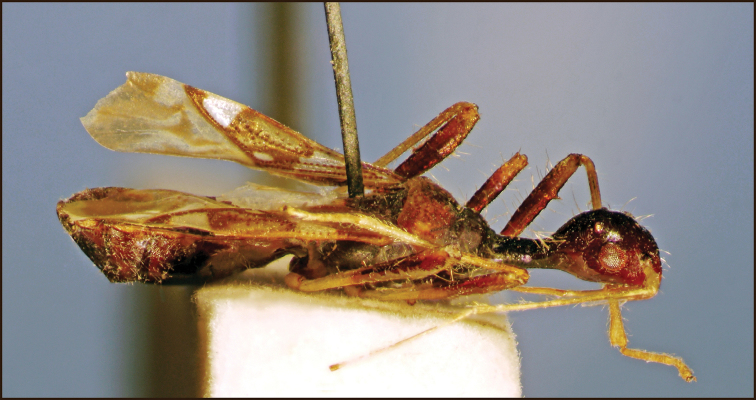
*Vertomannus* (s. str.) *borneensis* sp. n., lateral view

Head globose, shiny, frons smooth, not wrinkled, not punctured (Fig. 3). Anteocular part shorter than postocular part without neck. Long hairs present on head and neck. Labium surpassing anterior margin of prosternum but ending before fore coxae, segment I reaching hind margin of eyes (Fig. 4).

Thorax: Pronotum trapezoidal, with long hairs, lateral margin concave, anterior lobe impunctate, narrower than posterior lobe which is punctured, posterior lobe a little more declivous than anterior lobe in lateral view. Scutellum longer than wide, sparsely punctured, with some long hairs.

Legs slender and long, fore femur with a single spine, provided with hairs which are longer than diameter of femur. Corium with two rows of punctures along claval suture and with irregular punctures on apical half of mesocorium, not pubescent. Membrane with four distinct veins, not forming cells.

Abdomen: densely pubescent with decumbent silvery hairs.

**Colouration.** Body fuscous. Posterior lobe of pronotum pale- brown except middle part, antenna pale. Bases of femora pale stramineous, apical halves brown, tibiae basally pale yellowish brown, gradually lightening towards apex, tarsi faint light brown. Scutellum with tiny stramineous spot at apex. Corium with extensive pale colouration, basal half pale except punctures, behind this area a wide stripe mostly ochraceous with brown spots and a tiny rounded white spot at apex of claval commissure; apex of corium brown with extensive white subapical spot. Membrane pale brownish, with some white spots.

##### Discussion.

*Vertomannus borneensis* sp. n. is smaller than the other described species of the genus. The rounded, shining anteocular part of head (without wrinkles) is diagnostic value. The long pilosity on head is more sparse than for other species. *Vertomannus crassus* Zheng, 1981 and *Vertomannus validus* Zheng, 1981 are clearly different in multispinose and thick fore femora and large globose anterior pronotal lobe. *Vertomannus brevicollum* Zheng, 1981 has a very short neck, shorter than postocular part, and the apical part of abdomen is yellowish brown. *Vertomannus capitatus*, *Vertomannus emeia* Zheng, 1981 and *Vertomannus ophiocephalus* Zheng, 1981 have a longer labium reaching the fore coxae and have at least two spines on fore femora. Additionally, *Vertomannus ophiocephalus* has a longer neck than the postocular part of head; and moderately shortened hemelytra, not reaching the end of the abdomen. The pale subapical spot of corium has concave fore margin at *Vertomannus capitatus*, however, *Vertomannus borneensis* has this spot with straight fore margin.

##### Etymology.

The name of the species refers on the type locality, “borneensis” is a Latin adjective in nominative case with meaning “origin from island Borneo” (now Kalimantan).

## Supplementary Material

XML Treatment for Vertomannus

XML Treatment for Vertomannus (Elongatomannus)

XML Treatment for Vertomannus (Elongatomannus) flavus

XML Treatment for Vertomannus (Vertomannus) borneensis
